# Brain Ultrastructure: Putting the Pieces Together

**DOI:** 10.3389/fcell.2021.629503

**Published:** 2021-02-18

**Authors:** Patrick C. Nahirney, Marie-Eve Tremblay

**Affiliations:** Division of Medical Sciences, University of Victoria, Victoria, BC, Canada

**Keywords:** electron microscopy, brain, neurons, glial cells, organelles, health, aging, disease

## Abstract

Unraveling the fine structure of the brain is important to provide a better understanding of its normal and abnormal functioning. Application of high-resolution electron microscopic techniques gives us an unprecedented opportunity to discern details of the brain parenchyma at nanoscale resolution, although identifying different cell types and their unique features in two-dimensional, or three-dimensional images, remains a challenge even to experts in the field. This article provides insights into how to identify the different cell types in the central nervous system, based on nuclear and cytoplasmic features, amongst other unique characteristics. From the basic distinction between neurons and their supporting cells, the glia, to differences in their subcellular compartments, organelles and their interactions, ultrastructural analyses can provide unique insights into the changes in brain function during aging and disease conditions, such as stroke, neurodegeneration, infection and trauma. Brain parenchyma is composed of a dense mixture of neuronal and glial cell bodies, together with their intertwined processes. Intracellular components that vary between cells, and can become altered with aging or disease, relate to the cytoplasmic and nucleoplasmic density, nuclear heterochromatin pattern, mitochondria, endoplasmic reticulum and Golgi complex, lysosomes, neurosecretory vesicles, and cytoskeletal elements (actin, intermediate filaments, and microtubules). Applying immunolabeling techniques to visualize membrane-bound or intracellular proteins in neurons and glial cells gives an even better appreciation of the subtle differences unique to these cells across contexts of health and disease. Together, our observations reveal how simple ultrastructural features can be used to identify specific changes in cell types, their health status, and functional relationships in the brain.

## Introduction

The first electron microscope was invented in 1931 by Max Knoll and Ernst Ruska (Savage et al., 2018). An electron microscope is a microscope that uses a beam of accelerated electrons as a source of illumination (Egerton, 2016). Because the wavelength of an electron can be up to 100,000 times shorter than visible light photons, electron microscopy (EM) has a higher resolving power than light microscopy, and can reveal the structure of much smaller objects. The original form of an electron microscope, the transmission electron microscope (TEM) uses an electron beam to create an image. A heated filament is used as a source of electrons. The beam is accelerated at high voltage, focused by electrostatic and electromagnetic lenses, and transmitted through an ultrathin (∼60–70 nm) specimen. As it goes through the specimen the beam carries information about its structure. The spatial variation in that information is afterward magnified by the objective lens system of the microscope which is then projected on a phosphor screen and captured by film or recorded with a charge-coupled device (CCD) camera (Knott and Genoud, 2013; Winey et al., 2014; Egerton, 2016).

To study the brain, for example in rodents, samples are normally fixed by transcardial perfusion of the animals with buffered aldehydes and then sliced to a 50–100 μm thickness using a vibratome. The vibratome sections are post-fixed with buffered osmium tetroxide (1–2%) which stabilizes membranes and enhances their contrast under the electron microscope. The sections are afterward embedded in a plastic resin, cut at 50–70 nm thickness with a diamond knife on an ultramicrotome, and collected onto grids, as required for imaging by TEM. Immunostaining with peroxidase, which produces an electron dense precipitate visible with EM, or with immunogold, can also be performed prior to embedding (Dykstra and Reuss, 2003; Skepper and Powell, 2008; Ligorio et al., 2009; Burry, 2010; Tremblay et al., 2010b; Savage et al., 2018). These steps need to be performed meticulously from brain sample fixation until plastic resin embedding to prevent ultrastructural degradation which would compromise the integrity of cellular membranes, organelles, and other subcellular elements (Dykstra and Reuss, 2003; Tremblay et al., 2010b; Bisht et al., 2016a; St-Pierre et al., 2019).

Over the last 60 years, ultrastructural examinations have provided important insights into the functional roles of neurons, synapses and glial cells under various conditions (Theodosis et al., 2008; Tremblay et al., 2010a; Paolicelli et al., 2011; Bourne and Harris, 2012; Schafer et al., 2012; Kettenmann et al., 2013; Knott and Genoud, 2013; Chung et al., 2015; Savage et al., 2018; Verkhratsky and Nedergaard, 2018). The neuronal, microglial, astrocytic as well as oligodendrocytic compartments can be identified using EM based on their different shape, size, nuclear heterochromatin pattern, organelles and cytoskeletal elements, as well as relationships with each other among the brain parenchyma. Plasma membranes, basement membranes, clefts in gap junctions, actin filaments, intermediate filaments, microtubules, ribosomes, extracellular spaces, glycogen granules, synaptic vesicles, dense-core vesicles, nuclear pores, and lysosomes, are only or best resolved with EM, at the highest resolution (reaching 1 nm) for a biological technique (Tremblay et al., 2010b; Savage et al., 2018; SynapseWeb, 2021). Although super-resolution microscopy, and more recently, expansion microscopy, were developed to resolve small structures, notably in correlation with EM (Carrier et al., 2020; Hoffman et al., 2020; Parra-Damas and Saura, 2020; Soria et al., 2020), the capacity of EM to reveal the ultrastructure of cells and their constituents without selective staining (although staining can be used to provide better visualization of membranes, cytoskeletal elements, and ribosomes, for instance; Dykstra and Reuss, 2003; Svitkina, 2009) confers an important advantage (Tremblay et al., 2010b; Savage et al., 2018).

Considering that only EM provides the resolution needed to reconstruct neuronal circuits completely with single-synapse information, EM with three-dimensional (3D) reconstruction is the main tool for the connectomics research, which aims to map the brain-wide circuits underlying behavior (Ohno et al., 2015; Swanson and Lichtman, 2016; Kubota et al., 2018). Several tools were developed in recent years to facilitate the acquisition, registration and segmentation which is the tracing of the elements of interest in all the pictures to generate 3D reconstructions (Knott and Genoud, 2013; Miranda et al., 2015; Savage et al., 2018; Carrier et al., 2020), allowing to identify and quantify organelles as well as cell types in the brain using deep machine learning analysis (Perez et al., 2014; García-Cabezas et al., 2016; Abdollahzadeh et al., 2019; Calì et al., 2019; Gómez-de-Mariscal et al., 2019; Santuy et al., 2020). Recent technological advances in scanning electron microscopy (SEM) have facilitated the automated acquisition of large tissue volumes in 3D at nanometer-resolution. Cutting-edge techniques, such as serial-block face and focused-ion beam SEM imaging (Knott and Genoud, 2013; Miranda et al., 2015; Savage et al., 2018, 2020; Carrier et al., 2020), combined with x-ray based modalities which allow to detect and correlate EM findings with disease hallmarks or neuroanatomical features in 3D, based on their electron density (Pacureanu et al., 2019; Töpperwien et al., 2020), will revolutionize the understanding of brain development, function, and plasticity across stages of the lifespan, regions, and contexts of health and disease.

This review article will summarize the series of identification criteria that we have developed in our EM studies to identify brain cells and their constituents, mainly within the cerebral cortex and the hippocampus of rodent models. Ultrastructural observations can provide thorough insights into the cellular and subcellular mechanisms underlying brain function and dysfunction in an unbiased manner, by revealing all cell types and their constituents simultaneously, without the restrictive use of markers that only show the elements of interest. Our goal with this review is to provide well-established and accessible resources that will help neuroscientists to identify biomarkers of aging and disease, including stroke, neurodegeneration, infection and trauma, as well as the outcome of various treatment strategies, with the use of EM. Our descriptions are based on the criteria defined by Peters et al. (Peters et al., 1990) and others (Skoff and Hamburger, 1974; Deitch and Banker, 1993; Fiala et al., 1998; SynapseWeb, 2021) that were refined over the years with the investigation of neuronal, microglial and astrocytic response to stress, aging, neurodegenerative disease pathology, and stroke, notably (Tremblay et al., 2010a, 2012; Nahirney et al., 2016; Henry et al., 2018; El Hajj et al., 2019; Savage et al., 2020). Our observations build on and complement the identification criteria that were historically established based on correlative light and EM (Luse, 1956; Herndon, 1964; Mori and Leblond, 1969; Griffin et al., 1972; Murabe and Sano, 1982; Shapiro et al., 2009). Emerging techniques that prevent aldehyde-fixation based artifacts, notably the reduction in extracellular space volume (Syková and Nicholson, 2008), such as cryo-EM, are beyond the scope of this review and covered elsewhere (Korogod et al., 2015; Subramaniam, 2019). Best practices in sample preparation to avoid hypoxia-induced artifacts are also discussed at length in excellent resources (Kuwajima et al., 2013).

## An Overview of the Ultrastructural Identification Criteria We Use

In the brain parenchyma, neurons can be dispersed or organized into layers depending on the brain region, while glial cells are generally dispersed, occupying satellite positions around neuronal cell bodies or interacting structurally with one another (Peters et al., 1990). Direct contacts between glial cell bodies, often taking place at the vasculature (Bardehle et al., 2013), can also suggest recent division events (Tremblay et al., 2012). While glial cells generally occupy non-overlapping territories (Bushong et al., 2002; Sousa et al., 2017), this organization can be lost upon neurological pathology, for instance epilepsy (Oberheim et al., 2008), and depends on visualization method (Stratoulias et al., 2019). Except where noted differently, the identification criteria summarized in this section are from Peters et al. (1990), SynapseWeb, and Fine Structure of the Aging Brain | Boston University.

### Cell Bodies

**FIGURE 1:**
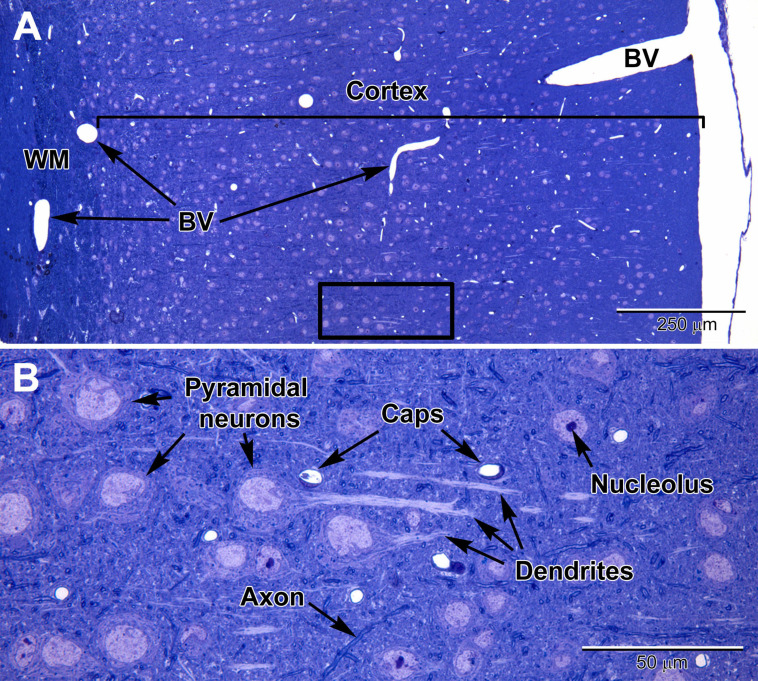
Light micrographs of a toluidine blue-stained plastic section from the adult mouse somatosensory brain region seen at low **(A)** and high **(B)** magnification. The cortex is arranged into layers that contain different types of neurons, with the largest neurons (pyramidal) in layers 4–6 which contain large euchromatic nuclei with nucleoli. Pyramidal neurons have prominent dendrites that project toward the outer cortex. At the surface of the cortex is a thin pia mater layer with underlying blood vessels (BV) that are present throughout the cortex. Deep to the cortex are myelinated axons of the white matter (WM). **(B)** At high magnification, capillaries (Caps) are evenly distributed in the brain parenchyma amongst the neurons, along with glial cells, which are less obvious, and contain smaller nuclei. Dispersed myelinated axons stain deeper blue and appear as wormlike structures in the parenchyma.

**FIGURE 2:**
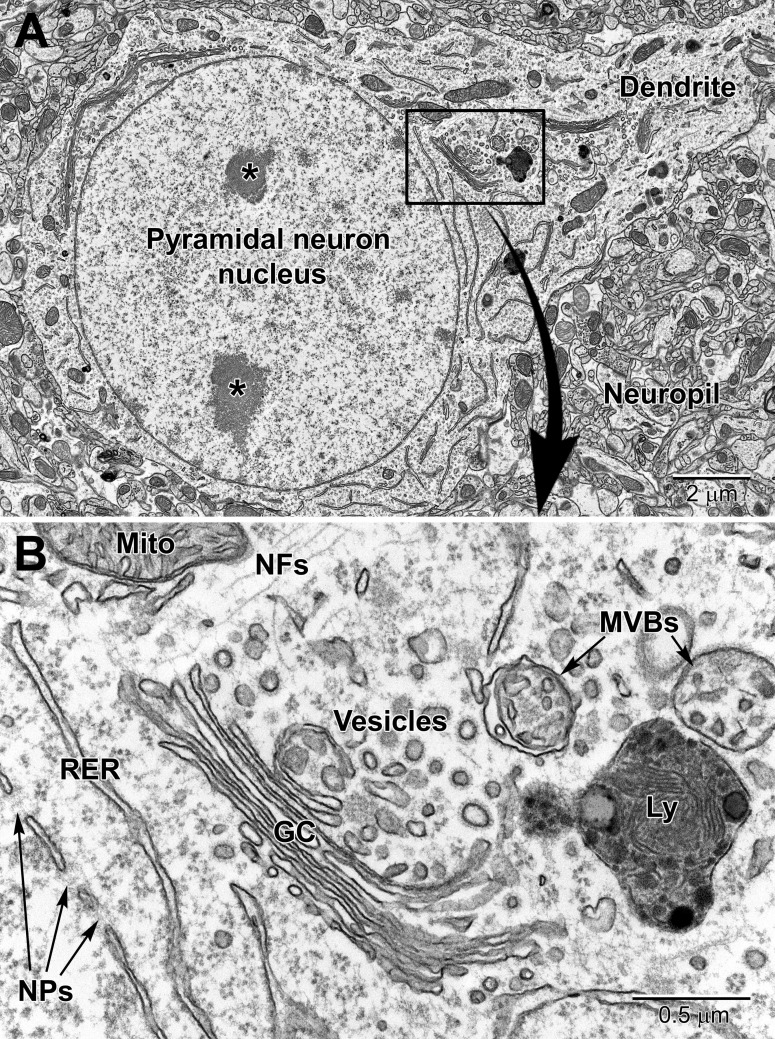
Low **(A)** and high **(B)** magnification EM images of a pyramidal neuron in layer 4/5 of the somatosensory cortex. The neuron contains a large euchromatic nucleus with two nucleoli (*) and a large primary dendrite emanates from the cell body. Surrounding the neuronal cell body is the neuropil consisting of a mixture of glial and neuronal processes, including synapses. The nuclear envelope contains numerous nuclear pores (NPs) and the perinuclear cytoplasm has a rich collection of organelles including mitochondria (Mito), RER, and Golgi complexes (GC) cisternae, vesicles, multivesicular bodies (MVBs), and lysosomes (Ly). Free ribosomes and neurofilaments (NFs) are dispersed in between the organelles.

**FIGURE 3:**
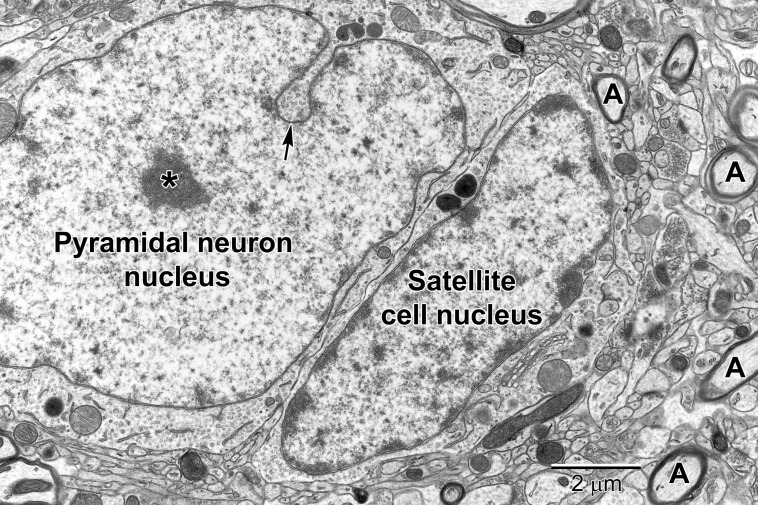
A pyramidal neuron in layer 5 with a satellite cell that displays ultrastructural features of an oligodendrocyte precursor cell closely abutting its cell body. The pyramidal neuron contains a large euchromatic nucleus with a centrally located nucleolus (*). Nuclear envelope invaginations (arrow) are occasionally seen in highly active neurons (Wittmann et al., 2009). Some neurons have intimately associated satellite cells (microglia and oligodendrocyte precursor cells) with a smaller ovoid nucleus. The surrounding neuropil contains synapses, axons (A) and glial cell processes.

Neurons can be identified in microscopic sections by their pale cytoplasm, a large and round euchromatic nucleus, as well as the presence of one or more electron dense nucleoli (Figures 1–3). Depending on the type of neuron, cell bodies can range dramatically in size. Typical pyramidal neurons in the deeper layers of the cortex range in size from ∼15 to 30 μm in diameter in rodents, and contain multiple cell processes including several dendrites traveling mostly toward the cortical surface and a single axon projecting deep toward the white matter. Small patches of heterochromatin are present in the nucleus, typically peripheral in location under the nuclear envelope which contains abundant nuclear pores (Figure 2B). Their surrounding cytoplasm, or perikaryon, contains an abundance of organelles that vary in shape and volume with the neuronal activity (Antón-Fernández et al., 2015; Tao-Cheng, 2018). A prominent Golgi complex(es) (Figure 2B) is/are present near the nucleus along with long flattened cisternae of rough endoplasmic reticulum (RER) studded with ribosomes (Peters et al., 1990). Free ribosomes, or groups of ribosomes (polyribosomes), occupy the interstitial spaces between organelles. Round to ovoid mitochondria are dispersed throughout the cytoplasm and occasional lysosomes filled with electron dense material (lipofuscin) are also present. Neurons contain an extensive array of neurofilaments and microtubules that extend into large dendrites emanating from the cell body. Multivesicular bodies, filled with small 40–80 nm sized vesicles, are also present near the Golgi complex, likely to be transported into the axon where they become concentrated at axon terminals (Peters et al., 1990).

**FIGURE 4:**
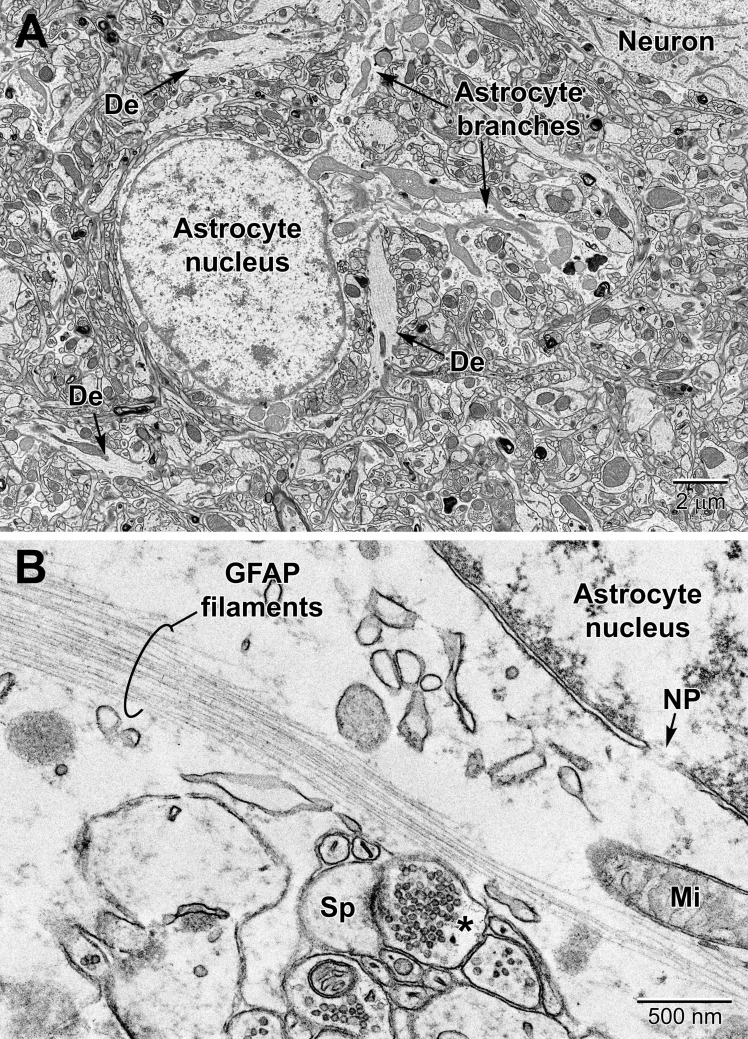
Protoplasmic astrocyte in the mouse cerebral cortex **(A)** and high magnification view of a fibrous astrocyte in the CA1 region of a rat hippocampus **(B)**. **(A)** In the cortex, astrocytes are branched and contain a relatively clear cytoplasm with a variety of organelles including mitochondria, RER, lysosomes, perinuclear Golgi complexes. In rodent astrocytes, mitochondria (Mi) stain relatively lighter after postfixation with reduced osmium. De, dendrite; Sp, dendritic spine; *, presynaptic terminal.

**FIGURE 5:**
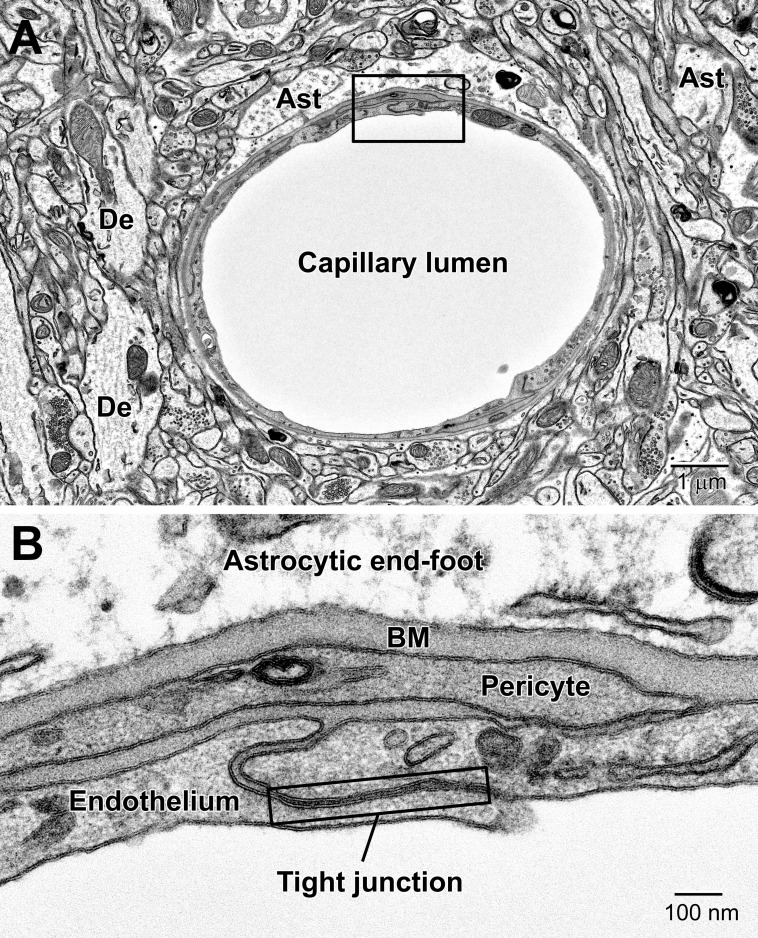
Components of the blood-brain barrier (BBB) and neurovascular unit. **(A)** Transverse section through a mouse cortical capillary showing the highly attenuated endothelium with small branches of pericytes sitting on their abluminal surface. **(B)** The BBB consists of the capillary endothelium with their tight junctions, underlying pericytes, both surrounded by a prominent amorphous basement membrane (BM) and astrocytic end-feet. End-feet connect with neighboring end-feet of other astrocytes via gap junctions. Ast, astrocyte; De, dendrite.

**FIGURE 6:**
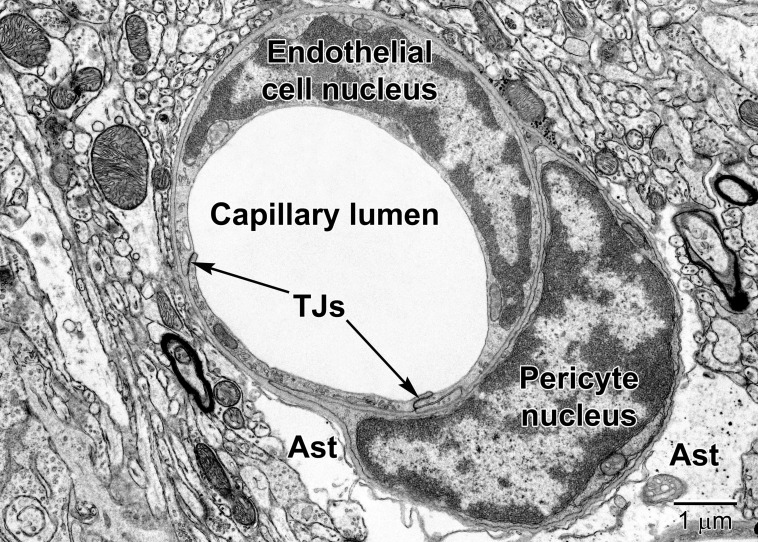
A fortuitous section of a mouse cortical capillary showing an endothelial cell and a pericyte at the level of their nuclei. Notice how the crescent-shaped heterochromatic nuclei take on the shape of the capillary. Tight junctions (TJs) link neighboring endothelial cells. The pericyte embraces the endothelium and resides within the same basement membrane. The position and branching nature of its processes are strategically situated to change the capillary lumen diameter. The relatively clear astrocyte end-feet (Ast) occupy the surrounding region around the capillary in the lower part of the image. Some electron dense glycogen granules are visible within the astrocyte.

Astrocytes, the largest and most populous of glial cells in the brain, are classified as either fibrous or protoplasmic. Elaborate and branched cell processes emanate from the stellate-shaped cell bodies into the neuropil. Protoplasmic astrocytes are recognized by their triangular shaped protuberances, pale nuclei with a thin rim of heterochromatin and pale irregular cytoplasm (Figure 4A and Supplementary Figure 1), often containing intermediate filaments (glial fibrillary acidic protein, GFAP) ranging in diameter from 8 to 12 nm (Figure 4B) (Peters et al., 1990). Astrocytes are distinguished by the relative sparseness of electron dense material in the cytoplasm. In addition, in our preparations with reduced osmium postfixation, we could identify astrocytic cell bodies and processes based on the fact that their mitochondrial membranes were less electron dense (lighter) than those found in neighboring neuronal and glial compartments (e.g., in neurons, dendrites, microglia, and oligodendrocytes) or in the endothelium and pericytes (Nahirney et al., 2016) (see Figure 4A and Supplementary Figure 1). This suggests that the membranes of astrocytic mitochondria contain a less dense concentration of lipids and/or proteins, which does not appear to be affected by the state of health or disease including stroke pathology (Nahirney et al., 2016). At the level of small blood vessels of the brain, astrocytes are an integral component of the blood-brain barrier (BBB) (Figures 5, 6). Expansions of astrocytic processes embrace capillaries and form so-called astrocytic (perivascular) end-feet. The BBB functions to protect neurons and glial cells in the CNS from drugs, toxins, as well as pro-inflammatory mediators and peripheral immune cells that would perturb the homeostasis, and includes in addition to astrocytes the microglia, which contribute to the *glia limitans* (Bisht et al., 2016c; Joost et al., 2019). Astrocytes, microglia, the endothelium and pericytes form together the neurovascular unit that regulates vascular remodeling and blood flow according to the needs of the neurons and glial cells (Andreone et al., 2015; Liu et al., 2020).

**FIGURE 7:**
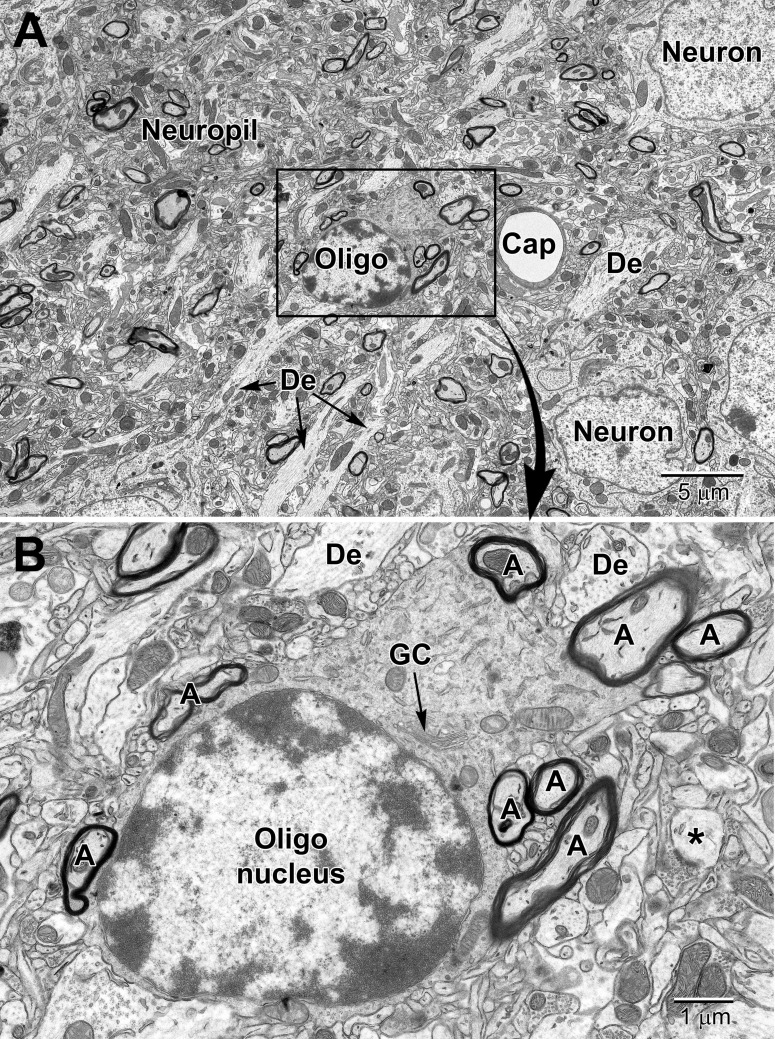
Low **(A)** and medium **(B)** magnification images of a typical oligodendrocyte (Oligo) sectioned at the level of its small heterochromatic nucleus in layer 5 of the mouse cortex. Several neurons are seen in the surrounding region for comparison of nuclear size and chromatin density. A neighboring capillary (Cap) and numerous dendrites (De) are seen in the surrounding neuropil. **(B)** A collection of myelinated axons **(A)** juxtapose and appear partially embedded within the relatively electron-dense cytoplasm of the oligodendrocyte. The dense cytoplasm contains a prominent perinuclear Golgi complex (GC), scattered mitochondria and small segments of rough endoplasmic reticulum. A spine head (*) synapses on a presynaptic terminal in the lower right of the image.

Compared with astrocytes, oligodendrocytes have a darker, electron-dense cytoplasm (Figure 7). They are mainly distinguished from neurons and other glial cells by their heterogeneous nuclear chromatin pattern, as well as squarish or rectangular-shape cytoplasm. Oligodendrocytes have short and wide endoplasmic reticulum cisternae organized in the vicinity of their nucleus, ribosomes, and wider space between nuclear membranes compared with microglia (Peters et al., 1990). The oligodendrocytic precursor cells look very different and sometimes occupy positions beside neurons (Figure 3). In many respects they resemble astrocytes. However, their nucleus is more irregular in shape. Their cytoplasm is pale, electron-lucent but they do not contain intermediate filaments nor extend processes making acute angles in the neuropil. Their stretches of endoplasmic reticulum are short, and their mitochondria are smaller than those of astrocytes. In addition, they do not accumulate lipidic inclusions during aging and in disease, even in old monkeys (Peters and Folger Sethares, 2020), contrary to neurons and other glial cells in the CNS (Tremblay et al., 2012).

**FIGURE 8:**
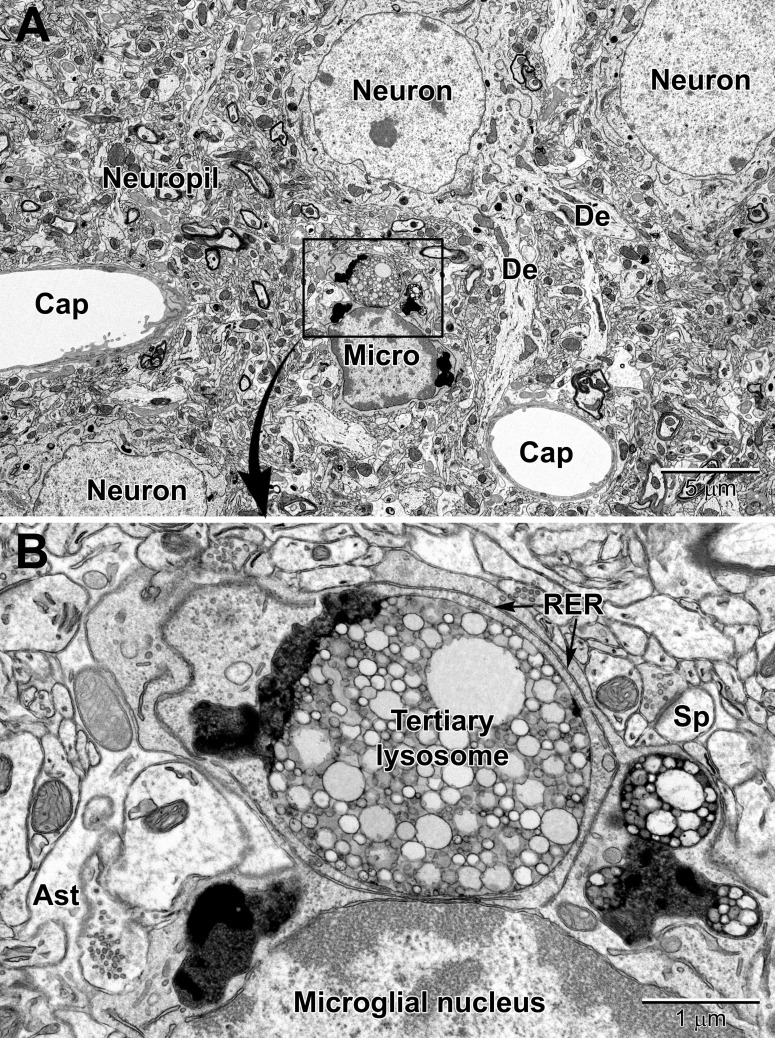
Low **(A)** and high **(B)** magnification views of a perivascular microglial cell in the cerebral cortex of an aged mouse (18 mo old). The nuclei of microglia are small and pleomorphic, and contain relatively more heterochromatin than neurons. Large, tertiary lysosomes with undigestible debris occupy the cytoplasm. Long stretches of rough endoplasmic reticulum (RER) characterize microglia that are active, in terms of producing inflammatory cytokines and other mediators. A dendritic spine (Sp) forms a synapse with a presynaptic terminal near the microglial cell, and an astrocytic branch (Ast) is in close proximity. Microglia are strategically situated between neurons and capillaries (Cap), and function as the resident immune cell and phagocyte required for maintaining brain health throughout life.

**FIGURE 9:**
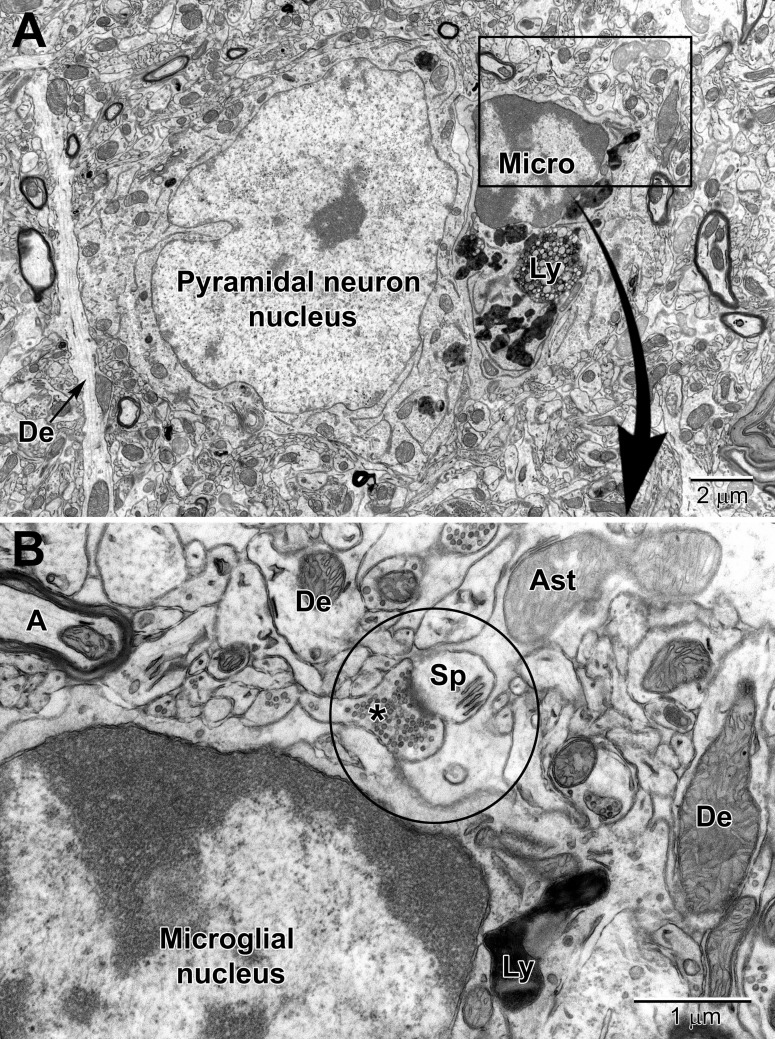
Low **(A)** and high **(B)** magnification views of a perineuronal microglial cell closely abutting a pyramidal neuron in the aged mouse cortex. Microglia are now believed to play an integral part in maintaining the synapse (aka quadpartite synapse, encircled) which includes the presynaptic terminal (*), dendritic spine (Sp), astrocyte process (Ast), and microglia. A, axon; De, dendrite; Ly, lysosome.

Similar to oligodendrocytes, microglia have a dark, electron-dense cytoplasm. Microglial cell bodies are recognized by their small size, frequent triangle shape, and the cheetah-pattern clumps of peripheral chromatin beneath their nuclear envelope and throughout their nucleoplasm (Tremblay et al., 2012; Savage et al., 2018). Examples of perivascular and perineuronal microglial cells are seen in Figures 8 and 9. The microglial cytoplasm often contains long stretches of endoplasmic reticulum cisternae and lipidic inclusions (i.e., lipofuscin, lipid bodies or droplets, and lysosomes; to be described in the *Organelles* section below) (Savage et al., 2018), which accumulate with aging. Microglia are frequently associated with pockets of extracellular space, contrary to other cell types in the mature healthy brain, and interact with both the vasculature and synapses (Tremblay et al., 2010a, 2012; Bisht et al., 2016c), as well as myelinated axons (Lampron et al., 2015; Bordeleau et al., 2020).

### Neuropil Elements

In the neuropil, which occupies most of the brain parenchyma outside of cell body layers and white matter, the intertwined neuronal and glial profiles can be identified according to criteria well defined previously (Skoff and Hamburger, 1974; Peters et al., 1990; Deitch and Banker, 1993; Harris and Weinberg, 2012), as summarized in Tremblay et al. (2007; 2009; 2010a; 2012).

**FIGURE 10:**
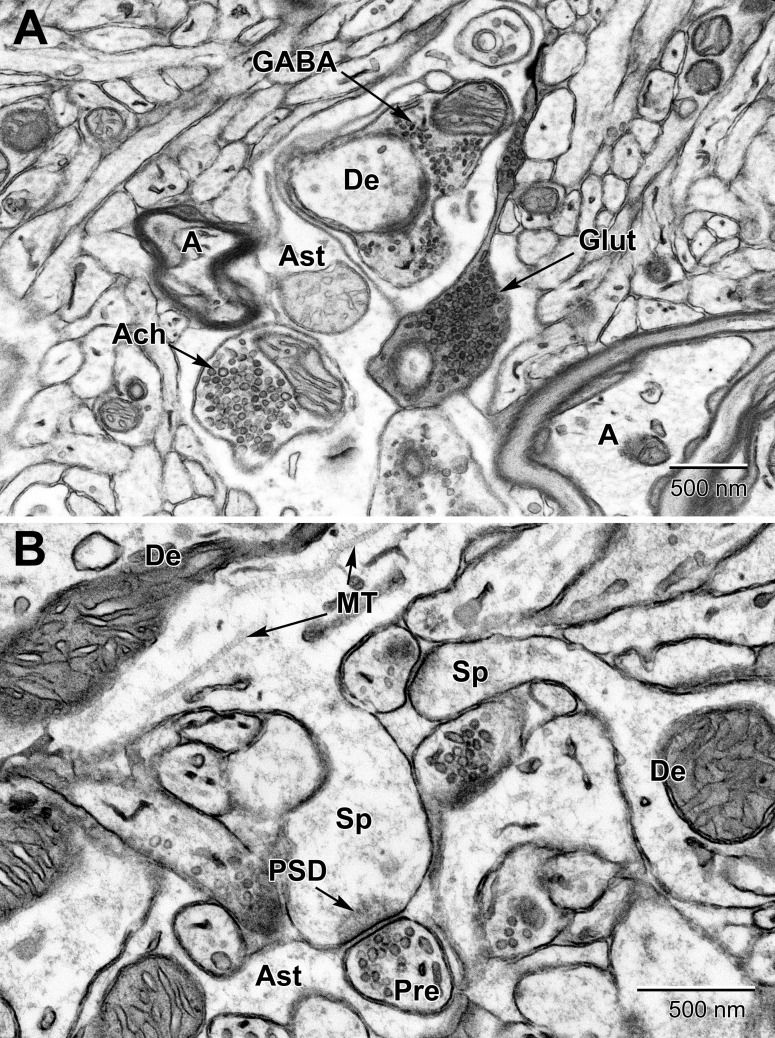
Examples of synapses in the mouse substantia nigra **(A)** and dentate gyrus **(B)**. **(A)** Three types of presynaptic terminals are evident in the substantia nigra: Cholinergic (Ach) with large (50–70 nm) vesicles, glutamatergic (Glut) with medium sized (35–50 nm) vesicles, and GABAergic (inhibitory) with small ovoid (20–35 nm) vesicles. **(B)** High magnification view of dendritic spines (Sp) emanating from dendrites (De) and synapsing with glutamatergic pre-synaptic terminals (Pre). post-synaptic densities (PSD) characterize glutamatergic synapses. Astrocytic processes (Ast) occupy the intervening spaces between these structures. Microtubules (MT) are seen running along the length of the dendrite. A, axon.

Dendritic branches are distinguished from unmyelinated axons by their more irregular contours, fewer microtubules, frequent protuberances (spines, filopodia, and small branches), and usual synaptic contacts with axon terminals (Tremblay et al., 2009). Dendritic spines display a characteristic “fluffy” or “cotton candy” type content due to their actin cytoskeleton (Papa et al., 1995). Spines (e.g., mushroom spines) may contain a spine apparatus, and receive frequent synaptic contacts from axon terminals (Figure 10). Their post-synaptic density, where receptors for neurotransmitters are located, is electron dense and visible without any immunostaining. Dendritic filopodia, which are considered to be immature spines (Berry and Nedivi, 2017), identified when seen protruding from dendritic branches, are distinguished from spines by their absence of a post-synaptic density, thinner neck, greater length, and pointed, rather than bulbous, head (Fiala et al., 1998). Unmyelinated axons are positively identified when they are found within fascicles or bundles of similar profiles. Axons becoming myelinated are also observed, either wrapped by oligodendrocytic processes with moderately dark cytoplasm or ensheathed by just a few turns of compact myelin and loose outer sheets (Tremblay et al., 2009). Axonal varicosities (also named “boutons”) correspond to enlarged portions of axons containing aggregated synaptic vesicles with neurotransmitters and frequently show “en passant” synaptic specializations, while axon terminals similarly display aggregated synaptic vesicles and synaptic specializations, but only at axonal extremities (Mechawar et al., 2000; Parent and Descarries, 2008; Tremblay et al., 2009).

In certain areas of the brain, such as the substantia nigra (Figure 10), up to three types of axon terminals can be identified by their vesicle diameter and shape (cholinergic, glutamatergic, and GABAergic) (Umbriaco et al., 1995; Mechawar et al., 2000; Ligorio et al., 2009). Synapses are identified by their synaptic cleft, i.e., direct apposition with less than 20 nm extracellular space between pre-synaptic axon terminals and post-synaptic dendritic spines or dendritic shafts, as revealed by tilt tomography (Supplementary Video 1) and more recently by focused ion-beam SEM in 3D (Savage et al., 2020). Only synaptic profiles presenting an unequivocal post-synaptic density are considered as asymmetric or excitatory in ultrathin section (Tremblay et al., 2007). Axonal growth cones are identified as considerable enlargements of axons, presenting a dark cytoplasm filled with large amounts of smooth endoplasmic reticulum and pleomorphic vesicles (Skoff and Hamburger, 1974; Peters et al., 1990; Deitch and Banker, 1993). They are distinguished from dendritic growth cones by their more frequent filopodial extensions, which often contact dendritic branches instead of axon terminals. Occasionally, these profiles are seen in direct continuity with axon terminals. Axonal filopodia are identified when extending from axonal growth cones. Nevertheless, distinction between axonal and dendritic growth cones or filopodia is not always obvious (Tremblay et al., 2009).

In health conditions, processes from protoplasmic astrocytes are recognized by their irregular and angular shapes, making acute angles as they go in-between the other elements of neuropil (Figure 4 and Supplementary Figure 1). In samples devoid of aldehyde-fixation artifacts (cryo-EM), astrocytic processes are more voluminous and make less acute angles around the other elements of neuropil, notably synapses (Korogod et al., 2015). By contrast, profiles from neurons and other types of glial cells (i.e., microglia and oligodendrocytic lineage cells) have a characteristic rounded shape. The processes from protoplasmic astrocytes frequently ensheath and can also phagocytose synapses, both pre-synaptic axon terminals and post-synaptic dendritic spines, within cellular inclusions during normal physiological conditions (Witcher et al., 2007; Theodosis et al., 2008; Chung et al., 2015; Verkhratsky and Nedergaard, 2018; Lee et al., 2020). Astrocytic processes have a clear, electron lucent cytoplasm, while oligodendrocytic and microglial processes have a moderately dense cytoplasm, and neurons have a cytoplasm showing intermediate gray levels between astrocytes and the other two glial cells (Peters et al., 1990). Oligodendrocytic processes display obtuse angles among the neuropil, similar to microglial processes, and are positively identified when their membrane is in direct continuity with myelinating or myelinated axons (Tremblay et al., 2009). However, it should be noted that microglial processes also interact with myelinated axons (Bordeleau et al., 2020).

Microglial processes display irregular contours with obtuse angles, a dense cytoplasm, numerous large vesicles, frequent endosomes and cellular inclusions (e.g., large lipidic vesicles, profiles of cellular membranes, and profiles of other structural elements including dendritic spines and axon terminals), as well as distinctive long stretches of endoplasmic reticulum (Tremblay et al., 2010a; El Hajj et al., 2019). They are typically surrounded by pockets of extracellular space that can vary in volume by two orders of magnitude (Tremblay et al., 2010a). These morphological characteristics of microglia were defined using immunocytochemical TEM against the marker ionized calcium binding adaptor molecule 1 (IBA1; Tremblay et al., 2010a) and allowed the identification of microglial processes in non-immunostained brain tissue, as confirmed using serial-section TEM (Tremblay et al., 2010a).

## An Overview of the Ultrastructural Changes Observed With Stress, Stroke, Aging and Disease

### Changes to Cell Bodies

With stress, aging and disease, darker cells are frequently observed within the brain parenchyma. The condensation state of the cytoplasm and nucleoplasm is associated with cellular shrinkage, considered a marker of cellular stress, and could explain this increased electron density (Bisht et al., 2016b).

Dark neurons display ultrastructural features of neurons, in terms of size, shape and organelles, as well as synaptic contacts (Peters and Folger Sethares, 2020). They are defined by their electron-dense cytoplasm and nucleoplasm, giving them a dark appearance under EM examination. They often display an accumulation of mitochondria and nuclear indentations (Tremblay et al., 2012) associated with structural remodeling and plasticity (Versaevel et al., 2014) or cellular stress, and various other markers of cellular stress (e.g., dilation of the endoplasmic reticulum and Golgi complex) (Henry et al., 2018). Dark cells were identified as putative oligodendrocytes by Dr. Alan Peters, based on their very close proximity to myelin sheaths. These cells contained autophagic vacuoles and small spherical bodies (Peters and Folger Sethares, 2020).

**FIGURE 11:**
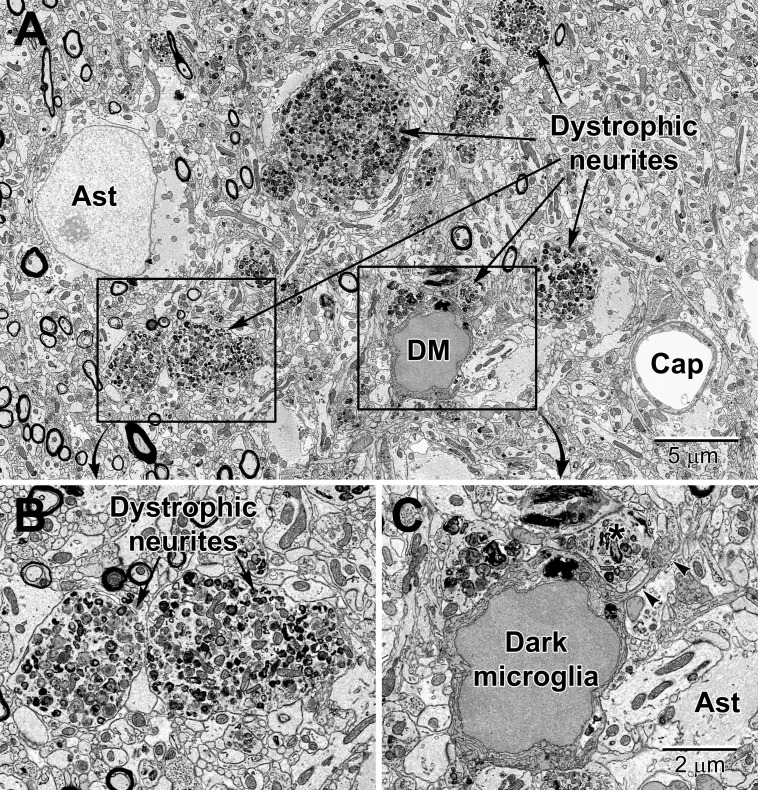
Dystrophic neurites closely surrounded by glial cells in the hippocampal CA1 region of a 20 mo old APP-PS1 mouse, a model of Alzheimer disease pathology **(A)**. Magnified regions are shown in panels **(B)** and **(C)**. An abnormally large astrocyte (Ast) contains distinctive intermediate filaments, while a dark microglia (DM) is recognized by the condensation state of its cytoplasm and nucleoplasm, in addition to its microglial features (e.g., long stretches of endoplasmic reticulum). **(C)** The dark microglia contains lipidic inclusions and extends a process (arrowheads) that contacts a synapse and encircles a dystrophic neurite (*). Cap, capillary.

Dark microglia display ultrastructural features of microglia, in terms of size, shape and organelles (e.g., long stretches of endoplasmic reticulum, associated pockets of extracellular space). They are, however, strikingly different from typical microglia due to their electron-dense cytoplasm and nucleoplasm, giving them a dark appearance in EM and their various markers of cellular stress (e.g., dilation of the endoplasmic reticulum and Golgi complex, alteration to mitochondrial ultrastructure, and loss of the microglial nuclear heterochromatin patterning). These cells frequently associate with the vasculature (see Supplementary Video 2), generally ensheathing the basement membrane while contributing to the *glia limitans* of capillaries (Bisht et al., 2016c). Another difference between the dark and typical microglia pertains to their synaptic interactions. Typical microglia rarely display processes directly protruding from their cell body in ultrathin section (Tremblay et al., 2010a), while dark microglia display several hyper-ramified processes, wrapping around instead of making focal contacts with synapses, and making acute angles in the neuropil (Bisht et al., 2016c; Hui et al., 2018a; St-Pierre et al., 2020). The contacted synapses include dystrophic neurites that are defined by their accumulation of autophagic vacuoles (Nixon, 2007) in Alzheimer disease pathology (Figure 11) (Bisht et al., 2016c). Dark microglia frequently contain endosomes with cellular elements such as axon terminals and dendritic spines which indicates a high phagocytic capacity and is suggestive of their specific involvement with the pathological remodeling of neuronal circuits (Bisht et al., 2016c).

Perivascular dark cells were also described recently, but it still remains undetermined whether these cells are dark microglia transiting from the parenchyma, or peripheral cells coming from the periphery (Bordeleau et al., 2020). These cells were identified by their markers of cellular stress, similar to dark microglia (Bisht et al., 2016c).

In addition, apoptotic cells, whether they are neuronal or glial, appear dark in EM (Bordeleau et al., 2020). They are recognized by their pyknotic nucleus, fragmentation and blebbing of the nuclear membrane, and accumulation of autophagosomes (see *Organelles* section for description) (Zhang et al., 1999).

### Changes to Cell Processes

**FIGURE 12:**
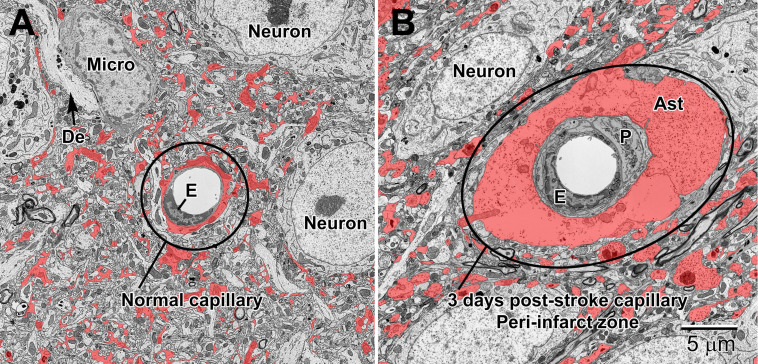
Normal **(A)** and 3 day post-stroke capillaries **(B)** in the peri-infarct zone of the mouse cortex. Astrocytes (shaded red) are pseudocolored to illustrate the drastic increase in volume after ischemia. The endothelium (E) and pericytes (P) are also enlarged after stroke. Note the accumulation of glycogen granules in the perivascular end-feet of the astrocytes. De, dendrite; Micro, microglial cell.

**FIGURE 13:**
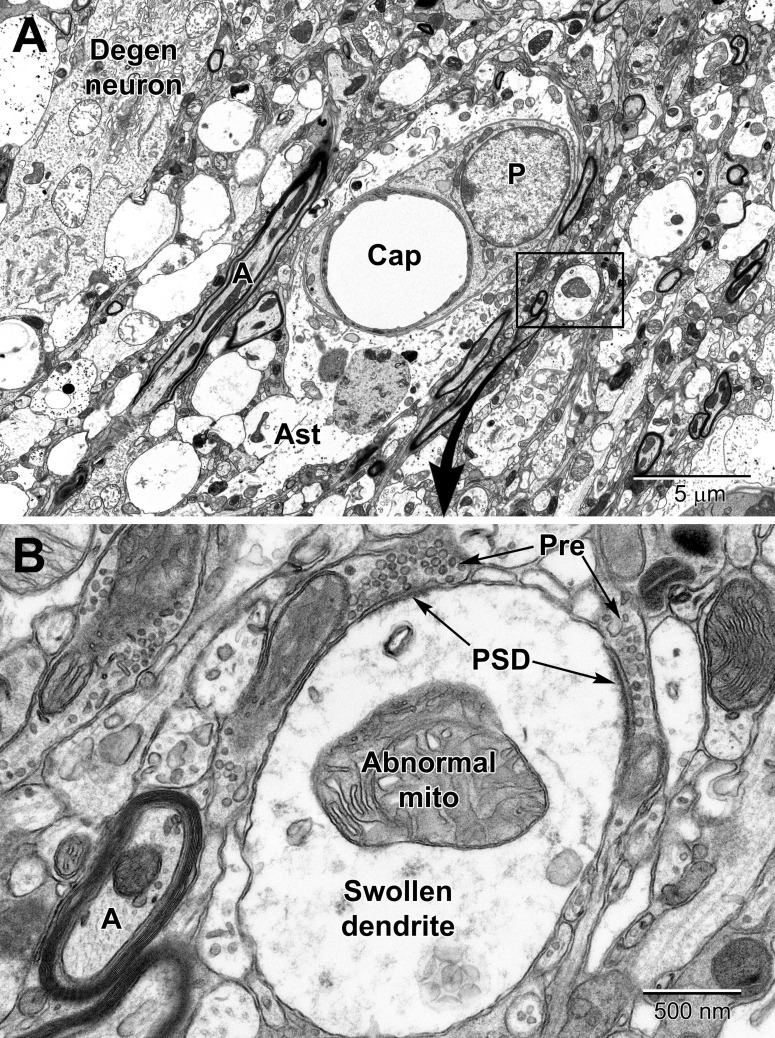
Low **(A)** and high **(B)** magnification views of changes to neurons and dendritic branches following ischemic stroke (3 days post-stroke) deep in the peri-infarct zone of the mouse cortex. A portion of a degenerating neuron (Degen neuron) is seen in the upper left and contains swollen mitochondria. Dendrites in the peri-infart zone swell and appear to absorb the spinous processes with evidence of the synapses apparent at the edges of the dendrite; note the post-synaptic densities (PSD) at the presynaptic terminal (Pre) contacts in **(A)**. Mitochondria in the swollen dendrite undergo dysplastic changes and exhibit dilated, loosely arranged cristae. Note the increase in size of the pericyte surrounding the capillary and the swollen astrocyte (Ast). Compare to a normal capillary in Figure 6.

Stroke is one of the main pathological conditions associated with apoptotic cell death to neurons, but it also involves swelling of glial cell processes in the brain parenchyma. Astrocytes in the peri-infarct zone respond to stroke by swelling and accumulating glycogen granules in their perivascular end-feet (Figures 12, 13). Together with the other cellular elements of the neurovascular unit (i.e., endothelium and pericytes), astrocytes become drastically enlarged after stroke (Nahirney et al., 2016) and this change is thought to be associated with their uptake of water, notably mediated via astrocytic aquaporin 4 channels, which was shown to modulate edema formation, as well as reflect a beneficial mechanism operating to minimize brain damage upon ischemia (Steiner et al., 2012; Stokum et al., 2015; Xiang et al., 2016). In addition, it was proposed that the astrocytic swelling during stroke may represent a beneficial response to BBB dysfunction, contributing to limiting the egress of plasma constituents and blood (hemorrhage) into the brain (Xiang et al., 2016). Our ultrastructural findings further revealed that the mechanisms causing BBB disruption upon stroke involve endothelial transport mediated via caveolae or vacuoles, instead of tight junction loss, and are associated with an increased thickness of the basement membrane (Nahirney et al., 2016). These effects of stroke on the BBB were diminished with normal aging (Nahirney et al., 2016), suggesting a reduced capacity of astrocytes and other cells of the neurovascular unit to cope with homeostatic challenges during aging. Biological aging is associated with reduced immunity and an increased risk of developing various disease conditions including the highly prevalent Alzheimer disease and stroke (Montecino-Rodriguez et al., 2013; Tay et al., 2017b).

In deeper regions of the peri-infarct region of an ischemic stroke (i.e., closer to the necrotic core), more drastic changes can be seen in astrocytic and neuronal processes including mitochondrial disruption and rupturing of membranes (Figure 13A). Axons and dendrites, and especially dendrites, show significant swelling in the peri-necrotic zone where they appear to lose their spinous processes (they get absorbed into the dendrite as it swells) as post-synaptic densities are commonly observed inside dendrites in contact with glutamatergic axon terminals (Figure 13B).

### Changes to Intracellular Elements

In neurons and glial cells, several markers of cellular stress or aging, as well as dystrophy, degeneration, and disease, can be identified ultrastructurally. The most frequently investigated ones are described below.

The best characterized marker of cellular stress is dilation of the endoplasmic reticulum and/or Golgi complex, which is associated with an accumulation of dysfunctional proteins. This feature is noted when the swelling between endoplasmic reticulum and/or Golgi cisternal membranes extends beyond 50 nm (Welch and Suhan, 1985; Schönthal, 2012; El Hajj et al., 2019). Autophagosomes are involved in autophagy – the removal of dysfunctional cellular components, and accumulate with cellular stress and aging (Leidal et al., 2018). They are observed in neurons and glial cells, and are identified by the presence of digested elements within endosomes enclosed by a double membrane (size ranging from 325 nm to 1.2 μm) (Hui et al., 2018a). Mitochondrial elongation, which is associated with mitochondrial stress, is noted in neurons and glial cells when their length exceeds 1 μm (Henry et al., 2018; Hui et al., 2018b; St-Pierre et al., 2019; Bordeleau et al., 2020). Lipofuscin granules, which are considered a hallmark of cellular aging, are identified in neurons and glial cells (except oligodendrocytic precursor cells; Tremblay et al., 2012) by their oval or round structure and finely granular composition endowed with a unique fingerprint-like pattern associated with their amorphous materials (Sohal and Wolfe, 1986; Henry et al., 2018). Lipid bodies, associated as well with cellular aging in glial cells, are further identified by their circular shape and homogenous core ranging from a size of 160 nm to 2.2 μm (Fujimoto et al., 2013; El Hajj et al., 2019).

In microglia, lysosomes – the organelles which fuse with endosomes to degrade cellular cargo during phagocytosis – are identified by their dense heterogeneous contents within a single membrane (De Duve, 1963; Holtzman et al., 1967; El Hajj et al., 2019). Primary lysosomes possess a homogenous granular content and their diameter ranges from 0.3 to 0.5 μm. Secondary lysosomes are 1 to 2 μm across, and their content is heterogeneous showing fusion with vacuoles. They are differentiated from primary lysosomes by their contacts with fusion endosomes. Tertiary lysosomes range in diameter between 1.5 and 2.5 μm, and they are usually fused to one or two vacuoles associated with lipofuscin granules, as well as lipidic inclusions showing signs of degradation (Figures 8, 9). Lipidic inclusions are identified as the clustering of round organelles with an electron dense, either opaque or limpid, cytoplasm enclosed by a single membrane (El Hajj et al., 2019; Bordeleau et al., 2020). A phagocytic index can be compiled by summing up the endosomes containing cellular materials such as membranes, axon terminals with 40 nm synaptic vesicles and dendritic spines with a postsynaptic density, on a microglial cell body or process basis (Milior et al., 2016; Lecours et al., 2020). The proportion of “gitter” cells, which are microglia filled with lipid bodies and cellular debris, can be determined by counting microglial cell bodies with more than four lipid bodies and at least one lipofuscin granule, or cells with at least one large lipid body and multiple lipofuscin granules (Tremblay et al., 2012).

### Changes to Extracellular Elements and Intercellular Relationships

In the vicinity of microglial cell bodies and processes, degradation activities (e.g., degenerating myelin and extracellular digestion) were found to be exacerbated with aging and disease. In particular, extracellular space pockets containing debris, which could result from “exophagy” (the degradation of cellular constituents by lysosomal enzymes released extracellularly), exocytosis (the process of expelling the contents of a membrane-bound vesicle into the extracellular space, often lysosomal and in preparation for phagocytosis (Haka et al., 2016), or pinocytosis (also named bulk-phase endocytosis, by which immune cells can take up extracellular contents in a non-phagocytic manner; Kruth, 2011) become more prevalent with Alzheimer disease pathology. These space pockets containing debris are defined by the appearance of degraded materials (including cellular membranes and organelles) or debris in the extracellular space nearby microglia (El Hajj et al., 2019). In contrast, degenerating myelin is recognized by ballooning, swelling or distancing of myelin sheaths (Peters et al., 1990; Bordeleau et al., 2020) and is more often observed in aging (Peters and Folger Sethares, 2020) and demyelinating diseases such as multiple sclerosis pathology (Lampron et al., 2015). In addition, the prevalence of microglial contacts with synaptic clefts, termed synaptic contacts, which has been shown to be altered in disease states, such as Huntington disease pathology, can be determined by counting direct appositions between microglial plasma membrane and synapses between pre-synaptic axon terminals (identified by their synaptic vesicles) and post-synaptic dendritic spines (post-synaptic density) (Savage et al., 2020). Microglia perform various types of functional interventions at synapses, including synaptic stripping, which is the physical separation of pre- and post-synaptic elements by their intervening processes (Trapp et al., 2007). Microglial active contribution as the fourth element of the quadpartite synapse (Figure 9) is associated with important roles in synapse formation, maturation, structural, and functional plasticity, as well as elimination throughout life (Tay et al., 2017a, b). Considering the recent findings on dark microglia, which make extensive interactions with synapses, even more than the typical microglia, an updated version including these cells as the fifth element of synapses is also proposed here.

## Conclusion and Perspective

Overall, this review provides a survey analysis of distinctive ultrastructural features that can inform on the state of health, stress, dystrophy or degeneration of the neurons and different types of glial cells (astrocytes, oligodendrocytes, and microglia) in the brain parenchyma (see Table 1 for summary). Comparing nanoscale information about cell bodies, processes, organelles and cytoskeletal elements, without selective staining to visualize the elements of interest, all at once and with the very best spatial resolution afforded by a biological technique (1 nm), reveals differences in their cellular function and dysfunction. The ultrastructural analysis becomes especially enlightening when comparing brain regions, stages of life, and contexts of health or disease, as well as sexes and species. The recent developments in the field of imaging have allowed to significantly increase the speed and automation of EM imaging acquisition, registration and segmentation, for both two-dimensional (2D) and 3D visualization (Miranda et al., 2015; Savage et al., 2018; Carrier et al., 2020), as well as organelle and cell type identification in the brain (Perez et al., 2014; García-Cabezas et al., 2016; Abdollahzadeh et al., 2019; Calì et al., 2019; Gómez-de-Mariscal et al., 2019; Santuy et al., 2020; among others). Recent breakthroughs further allowed researchers to image biological samples at a subatomic resolution and without any aldehyde fixation artifacts (e.g., cryo-EM; Subramaniam, 2019, named method of the year in 2016 by Nature Methods). In addition, various strategies were proposed for the efficient correlation of light and EM data, including with X-ray modalities (Begemann and Galic, 2016; Pacureanu et al., 2019; Töpperwien et al., 2020). Together, these advancements are expected to tremendously enhance the possibilities of identifying biomarkers and validating treatment strategies with EM.

**TABLE 1:** Main ultrastructural identification criteria and pathological features of the parenchymal brain cells discussed in this review.

Type	Electron density	Structures and their features			Alterations
		*Cell bodies*		*Processes*	
		*Nucleus*	*Perikaryon*		
Neurons	Intermediate gray levels between astrocytes / oligodendrocyte precursors & mature oligodendrocytes / microglia	– Large and round euchromatic nucleus – Small patches of heterochromatin under the nuclear envelope – One or more electron dense nucleoli – Abundant nuclear pores	– Golgi complex(es) – Long flattened cisternae of rough ER – Free ribosomes and polyribosomes – Round to ovoid mitochondria – Occasional lysosomes with lipofuscin – Neurofilaments and microtubules – Multivesicular bodies	– Dendrites: irregular contours, few microtubules, frequent protrusions (spines, filopodia, small branches) – Spines: Bulbous head, fluffy’ or ‘cotton candy’ content, axonal input, spine apparatus (ER), post-synaptic density – Filopodia: Absence of post-synaptic density, thin neck, long length, pointed head – Unmyelinated axons: Found within fascicles or bundles of similar profiles – Myelinated axons: Ensheathed by compact myelin – Axonal varicosities (or ‘boutons’): axonal enlargements with aggregated synaptic vesicles & ‘en passant’ synaptic specializations – Axon terminals: axonal extremities with aggregated synaptic vesicles and synaptic specializations – Synapses: direct apposition between pre-synaptic axon terminals and post-synaptic dendritic spines or shafts	– Dark neurons: electron-dense cytoplasm and nucleoplasm, accumulation of mitochondria, nuclear indentations, and other markers of cellular stress (e.g., ER and Golgi dilation) Apoptotic neurons: electron-dense, pyknotic nucleus, fragmentation and blebbing of nuclear membrane, accumulation of autophagosomes Swollen dendrites: loss of dendritic spines Swollen axons
Astrocytes	Pale	– Thin rim of heterochromatin	– Stellate-shaped – Irregular contours – Frequent intermediate filaments – Sparseness of electron dense material – Mitochondria with lighter membranes than other cell types	– Irregular and angular shapes contrary to other glial processes (except dark microglia) – Frequently ensheath synapses, making direct contacts with pre-synaptic axon terminals, synaptic clefts, and post-synaptic elements (spines, dendrites), and neuronal cell bodies – Can contain phagocytosed synapses (both pre-synaptic axon terminals and post-synaptic dendritic spines) within endosomes	– Reactive astrocytes: abundant intermediate filaments, swelling and accumulation of glycogen granules in perivascular end-feet, mitochondrial disruption
Mature oligodendrocytes	Dark	– Heterogeneous heterochromatin pattern	– Squarish or rectangular-shaped – Short and wide ER cisternae – Ribosomes – Wider space between nuclear membranes than for microglia	– Obtuse angles in the neuropil – Positively identified when their plasma membrane is in direct continuity with myelinated axons	– Dark oligo-dendrocytes: cellular stress markers (ER and Golgi dilation), autophagosomes, elongated mitochondria, lipofuscin and other lipidic inclusions
Oligodendrocyte precursors	Pale	– Thin rim of heterochromatin	– Pale cytoplasm – Devoid of intermediate filaments – Short ER – Small mitochondria – Satellite positions	– Obtuse angles in the neuropil	– Do not accumulate lipidic inclusions during aging and in disease, contrary to other cell types
Microglia	Dark	– Small size – Triangle-shaped – Cheetah-pattern clumps of heterochromatin beneath the nuclear envelope and throughout the nucleoplasm	– Often contains long stretches of ER cisternae – Frequent lipidic inclusions (i.e., lipofuscin, lipid bodies or droplets, lysosomes) – Associated pockets of extracellular space, contrary to other cell types in the mature healthy brain – Frequent contacts with synapses (pre-synaptic axon terminals, synaptic clefts, post-synaptic dendrites or spines) – Satellite positions	– Irregular contours with obtuse angles (except for the dark microglia which make acute angles in the neuropil) – Frequent contacts with synapses (pre-synaptic axon terminals, synaptic clefts, post-synaptic dendrites and spines) and neuronal cell bodies – Frequent contacts with other glial cells – Numerous large vesicles and endosomes – Frequent cellular inclusions (e.g., large lipidic vesicles, cellular membranes, myelin, and profiles of other structural elements including pre-synaptic axon terminals and post-synaptic dendritic spines) – Distinctive long stretches of ER cisternae, contrary to the other glial cell types – Associated pockets of extracellular space, contrary to other cell types in the mature healthy brain	– Dark microglia: stress markers (e.g., dilated ER and Golgi, alteration to mitochondrial ultrastructure), loss of microglial nuclear heterochromatin patterning, frequent association with the vasculature, extensive synaptic interactions – Both typical and dark microglia can display changes in phagocytosis, exophagy, and synaptic contacts with pathology

*ER, endoplasmic reticulum.*

## Author Contributions

PN and M-ET designed and wrote the review manuscript. PN prepared the figures.

## Conflict of Interest

The authors declare that the research was conducted in the absence of any commercial or financial relationships that could be construed as a potential conflict of interest.

## Abbreviations

BBB: blood-brain barrier
CCD: charge-coupled device
EM: electron microscopy
GFAP: glial fibrillary acidic protein
IBA1: ionized calcium binding adaptor molecule 1
PSD: post-synaptic density
SEM: scanning electron microscopy
TEM: transmission electron microscopy
2D: two-dimensional
3D: three-dimensional

## References

[B1] Abdollahzadeh A. Belevich I. Jokitalo E. Sierra A. Tohka J. (2019). DeepACSON: automated segmentation of white matter in 3D electron microscopy. *bioRxiv*[Preprint]. 10.1101/828541 PMC7876004 33568775

[B2] Andreone B. J. Lacoste B. Gu C. (2015). Neuronal and vascular interactions. *Annu. Rev. Neurosci.* 38 25–46. 10.1146/annurev-neuro-071714-033835 25782970 PMC5729758

[B3] Antón-Fernández A. León-Espinosa G. DeFelipe J. Muñoz A. (2015). Changes in the golgi apparatus of neocortical and hippocampal neurons in the hibernating hamster. *Front. Neuroanat.* 9:157. 10.3389/fnana.2015.00157 26696838 PMC4678224

[B4] Bardehle S. Krüger M. Buggenthin F. Schwausch J. Ninkovic J. Clevers H. (2013). Live imaging of astrocyte responses to acute injury reveals selective juxtavascular proliferation. *Nat. Neurosci.* 16 580–586. 10.1038/nn.3371 23542688

[B5] Begemann I. Galic M. (2016). Correlative light electron microscopy: connecting synaptic structure and function. *Front. Synaptic Neurosci.* 8:28. 10.3389/fnsyn.2016.00028 27601992 PMC4993758

[B6] Berry K. P. Nedivi E. (2017). Spine dynamics: are they all the same? *Neuron* 96 43–55. 10.1016/j.neuron.2017.08.008 28957675 PMC5661952

[B7] Bisht K. El Hajj H. Savage J. C. Sánchez M. G. Tremblay M. -È (2016a). Correlative light and electron microscopy to study microglial interactions with β-amyloid plaques. *J. Vis. Exp.* 112:54060. 10.3791/54060 27286292 PMC4927759

[B8] Bisht K. Sharma K. Lacoste B. Tremblay M. -È (2016b). Dark microglia: why are they dark? *Commun. Integr. Biol.* 9:e1230575. 10.1080/19420889.2016.1230575 28042375 PMC5193044

[B9] Bisht K. Sharma K. P. Lecours C. Sánchez M. G. El Hajj H. Milior G. (2016c). Dark microglia: a new phenotype predominantly associated with pathological states. *Glia* 64 826–839. 10.1002/glia.22966 26847266 PMC4949554

[B10] Bordeleau M. Lacabanne C. Fernández de Cossío L. Vernoux N. Savage J. C. González-Ibáñez F. (2020). Microglial and peripheral immune priming is partially sexually dimorphic in adolescent mouse offspring exposed to maternal high-fat diet. *J. Neuroinflamm.* 17:264. 10.1186/s12974-020-01914-1 32891154 PMC7487673

[B11] Bourne J. N. Harris K. M. (2012). Nanoscale analysis of structural synaptic plasticity. *Curr. Opin. Neurobiol.* 22 372–382. 10.1016/j.conb.2011.10.019 22088391 PMC3292623

[B12] Burry R. W. (2010). “Electron microscopic immunocytochemistry,” in *Immunocytochemistry: A Practical Guide for Biomedical Research*, ed. Burry R. W. (New York, NY: Springer), 175–189. 10.1007/978-1-4419-1304-3_15

[B13] Bushong E. A. Martone M. E. Jones Y. Z. Ellisman M. H. (2002). Protoplasmic astrocytes in CA1 stratum radiatum occupy separate anatomical domains. *J. Neurosci.* 22 183–192. 10.1523/jneurosci.22-01-00183.2002 11756501 PMC6757596

[B14] Calì C. Agus M. Kare K. Boges D. J. Lehväslaiho H. Hadwiger M. (2019). 3D cellular reconstruction of cortical glia and parenchymal morphometric analysis from serial block-face electron microscopy of juvenile rat. *Prog. Neurobiol.* 183:101696. 10.1016/j.pneurobio.2019.101696 31550514

[B15] Carrier M. Robert M. -È González Ibáñez F. Desjardins M. Tremblay M. -È (2020). Imaging the neuroimmune dynamics across space and time. *Front. Neurosci.* 14:903. 10.3389/fnins.2020.00903 33071723 PMC7539119

[B16] Chung W.-S. Allen N. J. Eroglu C. (2015). Astrocytes control synapse formation, function, and elimination. *Cold Spring Harb. Perspect. Biol.* 7:a020370. 10.1101/cshperspect.a020370 25663667 PMC4527946

[B17] De Duve C. (1963). The lysosome. *Sci. Am.* 208 64–72. 10.1038/scientificamerican0563-64 14025755

[B18] Deitch J. S. Banker G. A. (1993). An electron microscopic analysis of hippocampal neurons developing in culture: early stages in the emergence of polarity. *J. Neurosci.* 13 4301–4315. 10.1523/JNEUROSCI.13-10-04301.1993 8410189 PMC6576363

[B19] Dykstra M. J. Reuss L. E. (2003). *Biological Electron Microscopy: Theory, Techniques, and Troubleshooting*, 2nd Edn. Boston, MA: Springer US, 10.1007/978-1-4419-9244-4

[B20] Egerton R. F. (2016). *Physical Principles of Electron Microscopy: An Introduction to TEM, SEM, and AEM*, 2nd Edn. New York, NY: Springer International Publishing, 10.1007/978-3-319-39877-8

[B21] El Hajj H. Savage J. C. Bisht K. Parent M. Vallières L. Rivest S. (2019). Ultrastructural evidence of microglial heterogeneity in alzheimer’s disease amyloid pathology. *J. Neuroinflammation* 16:87. 10.1186/s12974-019-1473-9 30992040 PMC6469225

[B22] Fiala J. C. Feinberg M. Popov V. Harris K. M. (1998). Synaptogenesis via dendritic filopodia in developing hippocampal area CA1. *J. Neurosci.* 18 8900–8911. 10.1523/JNEUROSCI.18-21-08900.1998 9786995 PMC6793554

[B23] Fujimoto T. Ohsaki Y. Suzuki M. Cheng J. (2013). Imaging lipid droplets by electron microscopy. *Methods Cell Biol.* 116 227–251. 10.1016/B978-0-12-408051-5.00012-7 24099296

[B24] García-Cabezas M. Á John Y. J. Barbas H. Zikopoulos B. (2016). Distinction of neurons, glia and endothelial cells in the cerebral cortex: an algorithm based on cytological features. *Front. Neuroanat.* 10:107. 10.3389/fnana.2016.00107 27847469 PMC5088408

[B25] Gómez-de-Mariscal E. Maška M. Kotrbová A. Pospíchalová V. Matula P. Muñoz-Barrutia A. (2019). Deep-learning-based segmentation of small extracellular vesicles in transmission electron microscopy images. *Sci. Rep.* 9:13211. 10.1038/s41598-019-49431-3 31519998 PMC6744556

[B26] Griffin R. Illis L. S. Mitchell J. (1972). Identification of neuroglia by light and electronmicroscopy. *Acta Neuropathol.* 22 7–12. 10.1007/BF00687546 4116350

[B27] Haka A. S. Barbosa-Lorenzi V. C. Lee H. J. Falcone D. J. Hudis C. A. Dannenberg A. J. (2016). Exocytosis of macrophage lysosomes leads to digestion of apoptotic adipocytes and foam cell formation. *J. Lipid Res.* 57 980–992. 10.1194/jlr.M064089 27044658 PMC4878183

[B28] Harris K. M. Weinberg R. J. (2012). Ultrastructure of synapses in the mammalian brain. *Cold Spring Harb. Perspect. Biol.* 4:a005587. 10.1101/cshperspect.a005587 22357909 PMC3331701

[B29] Henry M. S. Bisht K. Vernoux N. Gendron L. Torres-Berrio A. Drolet G. (2018). Delta opioid receptor signaling promotes resilience to stress under the repeated social defeat paradigm in mice. *Front. Mol. Neurosci.* 11:100. 10.3389/fnmol.2018.00100 29681795 PMC5897549

[B30] Herndon R. M. (1964). The fine structure of the rat cerebellum. II. The stellate neurons, granule cells, and glia. *J. Cell Biol.* 23 277–293. 10.1083/jcb.23.2.277 14222815 PMC2106521

[B31] Hoffman D. P. Shtengel G. Xu C. S. Campbell K. R. Freeman M. Wang L. (2020). Correlative three-dimensional super-resolution and block-face electron microscopy of whole vitreously frozen cells. *Science* 367:eaaz5357. 10.1126/science.aaz5357 31949053 PMC7339343

[B32] Holtzman E. Novikoff A. B. Villaverde H. (1967). Lysosomes and gerl in normal and chromatolytic neurons of the rat ganglion nodosum. *J. Cell Biol.* 33 419–435. 10.1083/jcb.33.2.419 4292314 PMC2108357

[B33] Hui C. W. St-Pierre A. El Hajj H. Remy Y. Hébert S. S. Luheshi G. N. (2018a). Prenatal immune challenge in mice leads to partly sex-dependent behavioral. Microglial, and molecular abnormalities associated with schizophrenia. *Front. Mol. Neurosci.* 11:13. 10.3389/fnmol.2018.00013 29472840 PMC5809492

[B34] Hui C. W. St-Pierre M.-K. Detuncq J. Aumailley L. Dubois M.-J. Couture V. (2018b). Nonfunctional mutant Wrn protein leads to neurological deficits, neuronal stress, microglial alteration, and immune imbalance in a mouse model of Werner syndrome. *Brain Behav. Immun.* 73 450–469. 10.1016/j.bbi.2018.06.007 29908963

[B35] Joost E. Jordão M. J. C. Mages B. Prinz M. Bechmann I. Krueger M. (2019). Microglia contribute to the glia limitans around arteries, capillaries and veins under physiological conditions, in a model of neuroinflammation and in human brain tissue. *Brain Struct. Funct.* 224 1301–1314. 10.1007/s00429-019-01834-8 30706162

[B36] Kettenmann H. Kirchhoff F. Verkhratsky A. (2013). Microglia: new roles for the synaptic stripper. *Neuron* 77 10–18. 10.1016/j.neuron.2012.12.023 23312512

[B37] Knott G. Genoud C. (2013). Is EM dead? *J. Cell Sci.* 126 4545–4552. 10.1242/jcs.124123 24124192

[B38] Korogod N. Petersen C. C. Knott G. W. (2015). Ultrastructural analysis of adult mouse neocortex comparing aldehyde perfusion with cryo fixation. *eLife* 4:e05793. 10.7554/eLife.05793 26259873 PMC4530226

[B39] Kruth H. S. (2011). Receptor-independent fluid-phase pinocytosis mechanisms for induction of foam cell formation with native LDL particles. *Curr. Opin. Lipidol.* 22 386–393. 10.1097/MOL.0b013e32834adadb 21881499 PMC4174540

[B40] Kubota Y. Sohn J. Kawaguchi Y. (2018). Large volume electron microscopy and neural microcircuit analysis. *Front. Neural Circuits* 12:98. 10.3389/fncir.2018.00098 30483066 PMC6240581

[B41] Kuwajima M. Mendenhall J. M. Harris K. M. (2013). Large-volume reconstruction of brain tissue from high-resolution serial section images acquired by SEM-based scanning transmission electron microscopy. *Methods Mol. Biol.* 950 253–273. 10.1007/978-1-62703-137-0_15 23086880 PMC3716574

[B42] Lampron A. Larochelle A. Laflamme N. Préfontaine P. Plante M.-M. Sánchez M. G. (2015). Inefficient clearance of myelin debris by microglia impairs remyelinating processes. *J. Exp. Med.* 212 481–495. 10.1084/jem.20141656 25779633 PMC4387282

[B43] Lecours C. St-Pierre M.-K. Picard K. Bordeleau M. Bourque M. Awogbindin I. O. (2020). Levodopa partially rescues microglial numerical, morphological, and phagolysosomal alterations in a monkey model of Parkinson’s disease. *Brain Behav. Immun.* 90 81–96. 10.1016/j.bbi.2020.07.044 32755645

[B44] Lee J.-H. Kim J. Noh S. Lee H. Lee S. Y. Mun J. Y. (2020). Astrocytes phagocytose adult hippocampal synapses for circuit homeostasis. *Nature* 10.1038/s41586-020-03060-3 [Epub ahead of print]. 33361813

[B45] Leidal A. M. Levine B. Debnath J. (2018). Autophagy and the cell biology of age-related disease. *Nat. Cell Biol.* 20 1338–1348. 10.1038/s41556-018-0235-8 30482941

[B46] Ligorio M. Descarries L. Warren R. A. (2009). Cholinergic innervation and thalamic input in rat nucleus accumbens. *J. Chem. Neuroanat.* 37 33–45. 10.1016/j.jchemneu.2008.08.003 18773952

[B47] Liu L.-R. Liu J.-C. Bao J.-S. Bai Q.-Q. Wang G.-Q. (2020). Interaction of microglia and astrocytes in the neurovascular unit. *Front. Immunol.* 11:1024. 10.3389/fimmu.2020.01024 PMC7362712 32733433

[B48] Luse S. A. (1956). Electron microscopic observations of the central nervous system. *J. Biophys. Biochem. Cytol.* 2 531–542. 10.1083/jcb.2.5.531 13376632 PMC2223987

[B49] Mechawar N. Cozzari C. Descarries L. (2000). Cholinergic innervation in adult rat cerebral cortex: a quantitative immunocytochemical description. *J. Comp. Neurol.* 428 305–318. 10.1002/1096-9861(20001211)428:2<305::aid-cne9>3.0.co;2-y 11064369

[B50] Milior G. Lecours C. Samson L. Bisht K. Poggini S. Pagani F. (2016). Fractalkine receptor deficiency impairs microglial and neuronal responsiveness to chronic stress. *Brain Behav. Immun.* 55 114–125. 10.1016/j.bbi.2015.07.024 26231972

[B51] Miranda K. Girard-Dias W. Attias M. de Souza W. Ramos I. (2015). Three dimensional reconstruction by electron microscopy in the life sciences: an introduction for cell and tissue biologists. *Mol. Reprod. Dev.* 82 530–547. 10.1002/mrd.22455 25652003

[B52] Montecino-Rodriguez E. Berent-Maoz B. Dorshkind K. (2013). Causes, consequences, and reversal of immune system aging. *J. Clin. Invest.* 123 958–965. 10.1172/JCI64096 23454758 PMC3582124

[B53] Mori S. Leblond C. P. (1969). Identification of microglia in light and electron microscopy. *J. Comp. Neurol.* 135 57–79. 10.1002/cne.901350104 4181000

[B54] Murabe Y. Sano Y. (1982). Morphological studies on neuroglia. V. Microglial cells in the cerebral cortex of the rat, with special reference to their possible involvement in synaptic function. *Cell Tissue Res.* 223 493–506. 10.1007/BF00218471 6124316

[B55] Nahirney P. C. Reeson P. Brown C. E. (2016). Ultrastructural analysis of blood-brain barrier breakdown in the peri-infarct zone in young adult and aged mice. *J. Cereb. Blood Flow Metab.* 36 413–425. 10.1177/0271678X15608396 26661190 PMC4759675

[B56] Nixon R. A. (2007). Autophagy, amyloidogenesis and alzheimer disease. *J. Cell Sci.* 120 4081–4091. 10.1242/jcs.019265 18032783

[B57] Oberheim N. A. Tian G.-F. Han X. Peng W. Takano T. Ransom B. (2008). Loss of astrocytic domain organization in the epileptic brain. *J. Neurosci.* 28 3264–3276. 10.1523/JNEUROSCI.4980-07.2008 18367594 PMC6670598

[B58] Ohno N. Katoh M. Saitoh Y. Saitoh S. (2015). Recent advancement in the challenges to connectomics. *Microscopy* 65 97–107. 10.1093/jmicro/dfv371 26671942

[B59] Pacureanu A. Maniates-Selvin J. Kuan A. T. Thomas L. A. Chen C.-L. Cloetens P. (2019). Dense neuronal reconstruction through X-ray holographic nano-tomography. *bioRxiv*[Preprint]. 10.1101/653188 PMC8354006 32929244

[B60] Paolicelli R. C. Bolasco G. Pagani F. Maggi L. Scianni M. Panzanelli P. (2011). Synaptic pruning by microglia is necessary for normal brain development. *Science* 333 1456–1458. 10.1126/science.1202529 21778362

[B61] Papa M. Bundman M. Greenberger V. Segal M. (1995). Morphological analysis of dendritic spine development in primary cultures of hippocampal neurons. *J. Neurosci.* 15 1–11. 10.1523/JNEUROSCI.15-01-00001.1995 7823120 PMC6578316

[B62] Parent M. Descarries L. (2008). Acetylcholine innervation of the adult rat thalamus: distribution and ultrastructural features in dorsolateral geniculate, parafascicular, and reticular thalamic nuclei. *J. Comp. Neurol.* 511 678–691. 10.1002/cne.21868 18924144

[B63] Parra-Damas A. Saura C. A. (2020). Tissue clearing and expansion methods for imaging brain pathology in neurodegeneration: from circuits to synapses and beyond. *Front. Neurosci.* 14:914. 10.3389/fnins.2020.00914 33122983 PMC7571329

[B64] Perez A. J. Seyedhosseini M. Deerinck T. J. Bushong E. A. Panda S. Tasdizen T. (2014). A workflow for the automatic segmentation of organelles in electron microscopy image stacks. *Front. Neuroanat.* 8:126. 10.3389/fnana.2014.00126 25426032 PMC4224098

[B65] Peters A. Folger Sethares C. (2020). *Chapter 16 – Dark cCells» Fine Structure of the Aging Brain.* Available online at: https://www.bu.edu/agingbrain/chapter-16-dark-cells/ (accessed November 4, 2020)

[B66] Peters A. Palay S. L. Webster H. F. (1990). *The Fine Structure of the Nervous System: Neurons and Their Supporting Cells*, 3rd Edn. New York, NY: Oxford University Press.

[B67] Santuy A. Tomás-Roca L. Rodríguez J.-R. González-Soriano J. Zhu F. Qiu Z. (2020). Estimation of the number of synapses in the hippocampus and brain-wide by volume electron microscopy and genetic labeling. *Sci. Rep.* 10:14014. 10.1038/s41598-020-70859-5 32814795 PMC7438319

[B68] Savage J. C. Picard K. González-Ibáñez F. Tremblay M. -È (2018). A brief history of microglial ultrastructure: distinctive features, phenotypes, and functions discovered over the past 60 years by electron microscopy. *Front. Immunol.* 9:803. 10.3389/fimmu.2018.00803 29922276 PMC5996933

[B69] Savage J. C. St-Pierre M.-K. Carrier M. El Hajj H. Novak S. W. Sanchez M. G. (2020). Microglial physiological properties and interactions with synapses are altered at presymptomatic stages in a mouse model of huntington’s disease pathology. *J. Neuroinflamm.* 17:98. 10.1186/s12974-020-01782-9 32241286 PMC7118932

[B70] Schafer D. P. Lehrman E. K. Kautzman A. G. Koyama R. Mardinly A. R. Yamasaki R. (2012). Microglia sculpt postnatal neural circuits in an activity and complement-dependent manner. *Neuron* 74 691–705. 10.1016/j.neuron.2012.03.026 22632727 PMC3528177

[B71] Schönthal A. H. (2012). Endoplasmic reticulum stress: its role in disease and novel prospects for therapy. *Scientifica (Cairo)* 2012:857516. 10.6064/2012/857516 24278747 PMC3820435

[B72] Shapiro L. A. Perez Z. D. Foresti M. L. Arisi G. M. Ribak C. E. (2009). Morphological and ultrastructural features of Iba1-immunolabeled microglial cells in the hippocampal dentate gyrus. *Brain Res.* 1266 29–36. 10.1016/j.brainres.2009.02.031 19249294 PMC2677570

[B73] Skepper J. N. Powell J. M. (2008). Immunogold staining of epoxy resin sections for Transmission Electron Microscopy (TEM). *Cold Spring Harb. Protoc.* 2008:db.rot5015. 10.1101/pdb.prot5015 21356849

[B74] Skoff R. P. Hamburger V. (1974). Fine structure of dendritic and axonal growth cones in embryonic chick spinal cord. *J. Comp. Neurol.* 153 107–147. 10.1002/cne.901530202 4810722

[B75] Sohal R. S. Wolfe L. S. (1986). “Chapter 11 lipofuscin: characteristics and significance,” in *Progress in Brain Research Aging of the Brain and Alzheimer’s Disease*, eds Swaab D. F. Fliers E. Mirmiran M. Van Gool W. A. Van Haaren F. (Amsterdam: Elsevier), 171–183. 10.1016/S0079-6123(08)64304-6 3554350

[B76] Soria F. N. Miguelez C. Peñagarikano O. Tønnesen J. (2020). Current techniques for investigating the brain extracellular space. *Front. Neurosci.* 14:570750. 10.3389/fnins.2020.570750 PMC7591815 33177979

[B77] Sousa C. Biber K. Michelucci A. (2017). Cellular and molecular characterization of microglia: a unique immune cell population. *Front. Immunol.* 8:198. 10.3389/fimmu.2017.00198 28303137 PMC5332364

[B78] Steiner E. Enzmann G. U. Lin S. Ghavampour S. Hannocks M.-J. Zuber B. (2012). Loss of astrocyte polarization upon transient focal brain ischemia as a possible mechanism to counteract early edema formation. *Glia* 60 1646–1659. 10.1002/glia.22383 22782669

[B79] Stokum J. A. Kurland D. B. Gerzanich V. Simard J. M. (2015). Mechanisms of astrocyte-mediated cerebral edema. *Neurochem. Res.* 40 317–328. 10.1007/s11064-014-1374-3 24996934 PMC4284155

[B80] St-Pierre M.-K. Bordeleau M. Tremblay M. (2019). Visualizing dark microglia. *Methods Mol. Biol.* 2034 97–110. 10.1007/978-1-4939-9658-2_8 31392680

[B81] St-Pierre M.-K. Šimončičová E. Bögi E. Tremblay M. (2020). Shedding light on the dark side of the microglia. *ASN Neuro.* 12:1759091420925335. 10.1177/1759091420925335 PMC7249604 32443939

[B82] Stratoulias V. Venero J. L. Tremblay M. Joseph B. (2019). Microglial subtypes: diversity within the microglial community. *EMBO J.* 38:e101997. 10.15252/embj.2019101997 31373067 PMC6717890

[B83] Subramaniam S. (2019). The cryo-EM revolution: fueling the next phase. *IUCrJ* 6:1–2. 10.1107/S2052252519000277 30713697 PMC6327181

[B84] Svitkina T. (2009). Imaging cytoskeleton components by electron microscopy. *Methods Mol. Biol.* 586 187–206. 10.1007/978-1-60761-376-3_10 19768431 PMC2925411

[B85] Swanson L. W. Lichtman J. W. (2016). From cajal to connectome and beyond. *Annu. Rev. Neurosci.* 39 197–216. 10.1146/annurev-neuro-071714-033954 27442070

[B86] Syková E. Nicholson C. (2008). Diffusion in brain extracellular space. *Physiol. Rev.* 88 1277–1340. 10.1152/physrev.00027.2007 18923183 PMC2785730

[B87] SynapseWeb (2021). Available online at: https://synapseweb.clm.utexas.edu/home (accessed January 1, 2021).

[B88] Tao-Cheng J.-H. (2018). Stimulation-induced structural changes at the nucleus, endoplasmic reticulum and mitochondria of hippocampal neurons. *Mol. Brain* 11:44. 10.1186/s13041-018-0387-2 30049284 PMC6062868

[B89] Tay T. L. Béchade C. D’Andrea I. St-Pierre M.-K. Henry M. S. Roumier A. (2017a). Microglia gone rogue: impacts on psychiatric disorders across the lifespan. *Front. Mol. Neurosci.* 10:421. 10.3389/fnmol.2017.00421 29354029 PMC5758507

[B90] Tay T. L. Savage J. C. Hui C. W. Bisht K. Tremblay M. -È (2017b). Microglia across the lifespan: from origin to function in brain development, plasticity and cognition. *J. Physiol.* 595 1929–1945. 10.1113/JP272134 27104646 PMC5350449

[B91] Theodosis D. T. Poulain D. A. Oliet S. H. R. (2008). Activity-dependent structural and functional plasticity of astrocyte-neuron interactions. *Physiol. Rev.* 88 983–1008. 10.1152/physrev.00036.2007 18626065

[B92] Töpperwien M. van der Meer F. Stadelmann C. Salditt T. (2020). Correlative x-ray phase-contrast tomography and histology of human brain tissue affected by alzheimer’s disease. *NeuroImage* 210:116523. 10.1016/j.neuroimage.2020.116523 31935519

[B93] Trapp B. D. Wujek J. R. Criste G. A. Jalabi W. Yin X. Kidd G. J. (2007). Evidence for synaptic stripping by cortical microglia. *Glia* 55 360–368. 10.1002/glia.20462 17136771

[B94] Tremblay M.-E. Riad M. Bouvier D. Murai K. K. Pasquale E. B. Descarries L. (2007). Localization of EphA4 in axon terminals and dendritic spines of adult rat hippocampus. *J Comp. Neurol.* 501 691–702. 10.1002/cne.21263 17299751

[B95] Tremblay M.-E. Riad M. Chierzi S. Murai K. K. Pasquale E. B. Doucet G. (2009). Developmental course of EphA4 cellular and subcellular localization in the postnatal rat hippocampus. *J. Comp. Neurol.* 512 798–813. 10.1002/cne.21922 19086003

[B96] Tremblay M.-E. Riad M. Majewska A. (2010b). Preparation of mouse brain tissue for immunoelectron microscopy. *J. Vis. Exp.* 41:2021. 10.3791/2021 20689505 PMC3156065

[B97] Tremblay M. -È Lowery R. L. Majewska A. K. (2010a). Microglial interactions with synapses are modulated by visual experience. *PLoS Biol.* 8:e1000527. 10.1371/journal.pbio.1000527 21072242 PMC2970556

[B98] Tremblay M. -È Zettel M. L. Ison J. R. Allen P. D. Majewska A. K. (2012). Effects of aging and sensory loss on glial cells in mouse visual and auditory cortices. *Glia* 60 541–558. 10.1002/glia.22287 22223464 PMC3276747

[B99] Umbriaco D. Garcia S. Beaulieu C. Descarries L. (1995). Relational features of acetylcholine, noradrenaline, serotonin and GABA axon terminals in the stratum radiatum of adult rat hippocampus (CA1). *Hippocampus* 5 605–620. 10.1002/hipo.450050611 8646286

[B100] Verkhratsky A. Nedergaard M. (2018). Physiology of astroglia. *Physiol. Rev.* 98 239–389. 10.1152/physrev.00042.2016 29351512 PMC6050349

[B101] Versaevel M. Braquenier J.-B. Riaz M. Grevesse T. Lantoine J. Gabriele S. (2014). Super-resolution microscopy reveals LINC complex recruitment at nuclear indentation sites. *Sci. Rep.* 4:7362. 10.1038/srep07362 25482017 PMC4258653

[B102] Welch W. J. Suhan J. P. (1985). Morphological study of the mammalian stress response: characterization of changes in cytoplasmic organelles, cytoskeleton, and nucleoli, and appearance of intranuclear actin filaments in rat fibroblasts after heat-shock treatment. *J. Cell Biol.* 101 1198–1211. 10.1083/jcb.101.4.1198 3900086 PMC2113902

[B103] Winey M. Meehl J. B. O’Toole E. T. Giddings T. H. (2014). Conventional transmission electron microscopy. *Mol. Biol. Cell* 25 319–323. 10.1091/mbc.E12-12-0863 24482357 PMC3907272

[B104] Witcher M. R. Kirov S. A. Harris K. M. (2007). Plasticity of perisynaptic astroglia during synaptogenesis in the mature rat hippocampus. *Glia* 55 13–23. 10.1002/glia.20415 17001633

[B105] Wittmann M. Queisser G. Eder A. Wiegert J. S. Bengtson C. P. Hellwig A. (2009). Synaptic activity induces dramatic changes in the geometry of the cell nucleus: interplay between nuclear structure. Histone H3 phosphorylation, and nuclear calcium signaling. *J. Neurosci.* 29 14687–14700. 10.1523/JNEUROSCI.1160-09.2009 19940164 PMC6666017

[B106] Xiang J. Tang Y. Li C. Su E. J. Lawrence D. A. Keep R. F. (2016). Mechanisms underlying astrocyte endfeet swelling in stroke. *Acta Neurochir. Suppl.* 121 19–22. 10.1007/978-3-319-18497-5_4 26463917

[B107] Zhang J. Reedy M. C. Hannun Y. A. Obeid L. M. (1999). Inhibition of caspases inhibits the release of apoptotic bodies: Bcl-2 inhibits the Initiation of formation of apoptotic bodies in chemotherapeutic agent-induced apoptosis. *J. Cell Biol.* 145 99–108. 10.1083/jcb.145.1.99 10189371 PMC2148221

