# Supplementary material for: Brain Ultrastructure: Putting the Pieces Together
Source: Front Cell Dev Biol. 2021 Feb 18;9:629503. doi: 10.3389/fcell.2021.629503 (PMC7930431; doi:10.3389/fcell.2021.629503)
Supplement: Supplementary file 4 [file Data_Sheet_1.pdf]

frontiers

Brain ultrastructure: Putting the pieces together

Patrick C. Nahirney and Marie-Eve Tremblay
Division of Medical Sciences
University of Victoria, B.C., Canada

Corresponding author:
Patrick C. Nahirney
Division of Medical Sciences
University of Victoria
Victoria, B.C. Canada
Tel.: 250 853-3659
email: nahirney@uvic.ca

Supplemental Data

Supplemental figure:

1. High-resolution zoomable EM image of the mouse brain cortex showing the extent of astrocytes (shaded red) between neuronal processes and other glial cells. Astrocytes function to maintain the internal environment for neurons and glia. Two large pyramidal neurons are in the upper left and a capillary is in the center of the image. A typical microglia is in the lower left adjacent to a large dendrite. JPG, 11000 x 7616 pixels, 10 MB.

Supplemental videos:

1. EM tomography tilt series (+/- 25 degrees, 5 degree intervals) showing two spines (above, containing PSDs) synapsing with a glutamatergic presynaptic terminal filled with ~40 nm diameter vesicles. MP4 video, 1 MB.

2. FIB-SEM z stack video of the mouse hippocampus showing the difference between typical and dark microglia. Both of these resident immune cells are in close apposition to a capillary. Imaging was performed at a 14 nm resolution in x, y, and z planes. MP4 video, 20 MB.
